# Crystal and mol­ecular structure of jatrophane diterpenoid (2*R*,3*R*,4*S*,5*R*,7*S*,8*S*,9*S*,13*S*,14*S*,15*R*)-2,3,8,9-tetra­acet­oxy-5,14-bis­(benzo­yloxy)-15-hydroxy-7-(iso­butano­yloxy)jatropha-6(17),11(*E*)-diene

**DOI:** 10.1107/S205698901901541X

**Published:** 2019-11-19

**Authors:** Hequn Yang, Jiangyu Zhao, Samat Talipov, Lidiya Izotova, Haji Akber Aisa, Bakhtiyar Ibragimov

**Affiliations:** a Key Laboratory of Plant Resources and Chemistry of Arid Zones, Xinjiang Technical Institute of Physics and Chemistry, Chinese Academy of Science, Urumqi 830011, People’s Republic of China; b Institute of Bioorganic Chemistry, Academy of Sciences of Uzbekistan, H. Abdullaev Str, 83, Tashkent, 100125, Uzbekistan

**Keywords:** crystal structure, hydrogen bonding, jatrophane diterpene

## Abstract

The structure of the jatrophane diterpenoid (ES2), C_46_H_56_O_15_, has ortho­rhom­bic (*P*2_1_2_1_2_1_) symmetry. The absolute configuration in the crystal has been determined as 2*R*,3*R*,4*S*,5*R*,7*S*,8*S*,9*S*,13*S*,14*S*,15*R* [the Flack parameter is −0.06 (11)]. The mol­ecular structure features intra­molecular O—H⋯O and C—H⋯O hydrogen bonding. In the crystal, C—H⋯O hydrogen bonds link the mol­ecules into supra­molecular columns parallel to the *a* axis. One of the acet­oxy substituents is disordered over two orientations in a 0.826 (8):0.174 (8) ratio.

## Abstract 2

A jatrophane diterpenoid was isolated from the fructus of *Euphorbia sororia* and its structure and absolute configuration have been established by X-ray crystallographic analysis.

## Chemical context   

Macrocyclic diterpenes demonstrate a range of biological effects, including modulability of multidrug resistance, cytotoxicity, anti­proliferative, anti-inflammatory, and anti­microbial activities (Hohmann *et al.*, 2002 ▸; Shi *et al.*, 2008 ▸; Vasas & Hohmann, 2014 ▸). Jatrophane diterpenes, which possess fused five- and twelve-membered carbon rings are usually substituted by a variety of aryl and benzyl groups. The title compound ES2, a new type of jatrophane diterpenoid ester isolated from the fruits of *Euphorbia sororia* is widely used as a traditional Uyghur medicine in China (Lu *et al.*, 2014 ▸) and shows promising chemo-reversal abilities compared to verapamil (Hu *et al.*, 2018 ▸). ES2 has demonstrated cytotoxicity and anti-multidrug resistance activity in multidrug-resistant MCF-7/ADR breast cancer cells (Fang *et al.*, 2018 ▸). The structure of this compound has been determined by X-ray structure analysis and reported in the present article.
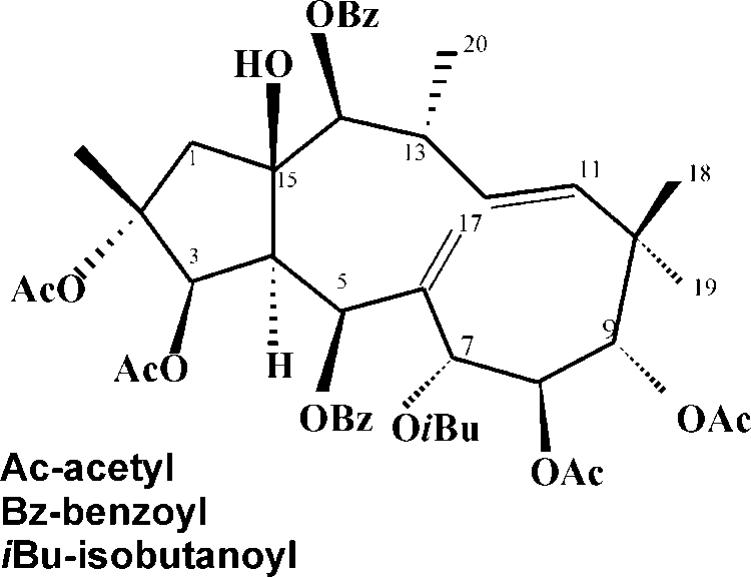


## Structural commentary   

**Figure 1:**
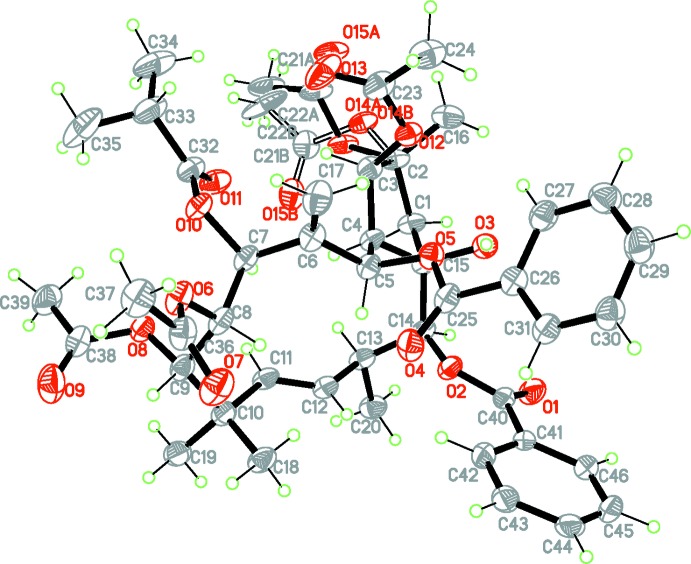
The mol­ecular structure of the title compound ES2 with atom labelling. Displacement ellipsoids are drawn at the 50% probability level.

The ES2 mol­ecule consists of five-membered and twelve-membered rings (Fig. 1 ▸). The configuration at the ring junction C4—C15 is *trans*. The five-membered ring adopts an envelope conformation, with atoms C1, C2, C3 and C4 coplanar to within 0.008 Å and atom C15 displaced from this mean plane by 0.631 (7) Å. The C11=C12 double bond and the C4—C5 and C7—C8 bonds in the twelve-membered ring adopt a *trans* conformation, with C10—C11—C12—C13 and C15—C4—C5—C6 torsion angles of 172.8 (4) and 178.7 (4)°, respectively. There are six asymmetric carbon atoms in the twelve-membered ring and four in the five-membered ring. This Jatrophane diterpene is substituted by a variety of functional groups. The benzo­yloxy substituents are both situated on the same side of the twelve-membered ring, but their conformation is not planar. The angles between the planes of the phenyl rings and the corresponding ester fragments is 15.50 (2)° (C5/O5/C25/O4/C26) and 10.00 (2)° (C14/O2/C40/O1/C41). Of the four acetyl substituents, two (at C3 and C8), as well as the hydroxyl at C15, are located on the same side as the benzoyl rings in relation to the twelve-membered ring.

Six intra­molecular hydrogen bonds, one conventional O—H⋯O and five weak C—H⋯O (Table 1 ▸), help to stabilize the mol­ecular structure. The O3—H3*A*⋯O5 hydrogen bond is formed between the 15-hy­droxy group as donor and the ether oxygen of the adjacent benzo­yloxy substituent.

## Supra­molecular features   

**Figure 2:**
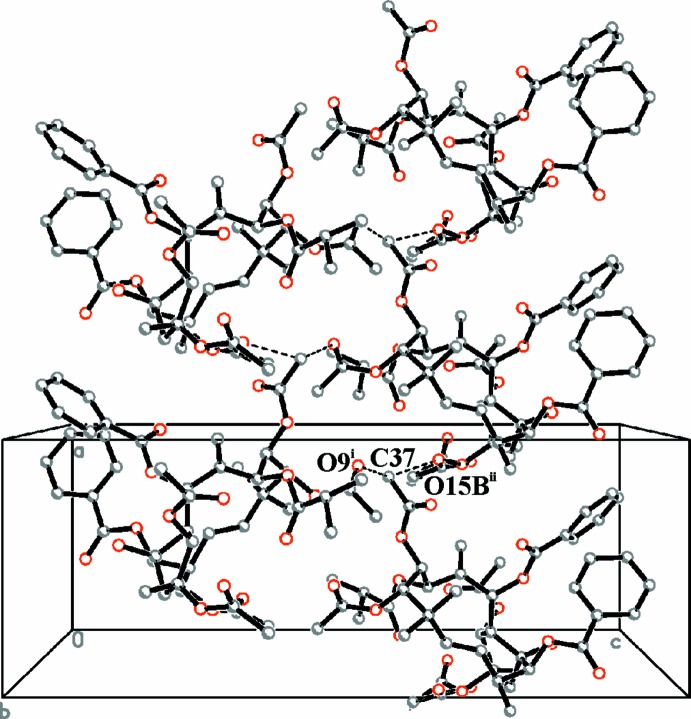
Crystal structure of the title compound ES2 viewed along the *b* axis. Inter­molecular hydrogen bonds (Table 1 ▸) are shown as dashed lines. Hydrogen atoms have been omitted for clarity. [Symmetry codes: (i) *x* + 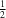, −*y* + 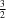, −*z* + 1; (ii) *x* + 1, *y*, *z*.]

In the crystal, two C—H⋯O hydrogen bonds form between the methyl group (C37) of the 8-acet­oxy group as donor and the carbonyl O atoms of the acet­oxy substituents in positions 2 (O15*B*) and 9 (O9) as acceptors (Table 1 ▸). These inter­actions link mol­ecules related by symmetry operation 2_1_ and translation parallel to the *a* axis, respectively. Together they form extended supra­mol­ecular columns parallel to the *a* axis (Fig. 2 ▸). Only van der Waals inter­actions occur between the columns. The OH group is not involved in inter­molecular hydrogen-bonding inter­actions, only intra­molecular.

## Database survey   

The Cambridge Structural Database (CSD version 5.40, last update November 2018; Groom *et al.*, 2016 ▸) includes crystallographic data for 19 jatrophane diterpenes. The first example of a similar compound in the literature, esulone A [(*E*)-(−)-(2*R*,3*R*,4*S*,5*R*,7*S*,8*R*,13*S*,15*R*)-diacet­oxy-5,7-dibenzo­yloxy-2,8-hy­droxy-jatropha-6(17),11-diene-9,14-dione (DEDMUU; Manners & Wong, 1985 ▸), was isolated from *Euphorbia esula* roots. Eight jatrophane esters were investigated by No­thias-Scaglia *et al.* (2014 ▸): JAQWUW, JAQXAD, YOLPOG, YOLPUM and YOLQAT, JAQXEH, JAQXIL and YOLQEX. Similar compounds studied are EZIHUS, EZIJAA and EZIJEE(Esposito *et al.*, 2016 ▸), PEMQON (Kar *et al.*, 1998 ▸), SUXHUO (Liu & Tan, 2001 ▸), and altotibetin A and altotibetin B (OKICIU and OKICOA; Li *et al.*, 2003 ▸). In ZUKLIA and ZUKLOG (terracinolide A and terracinolide B; Marco *et al.*, 1996 ▸) and ZELWEV01 (Hu *et al.*, 2018 ▸), a lactone ring substituent is present, so the configuration at the C5—C6 ring junction is *cis*.

The structure of EZIJAA, (2*R*,3*R*,4*S*,5*R*,7*R*,8*R*,9*R*,13*S*,15*R*)-2,9-diacet­oxy-3,8,15-trihy­droxy-5,7-dibenzo­yloxy-14-oxo­jatropha- 6(17),11(*E*)-diene diethyl ether solvate (Esposito *et al.*, 2016 ▸) is the most similar to that of the title compound. Both structures have *trans*-conjugated five- and twelve-membered rings, but the envelope conformation of the former in EZIJAA is different. Atom C4 (not C15 as in title structure) is out of the mean plane. In both structures, the substituent at C5 is a Bz-group, but in EZIJAA the benzyl ring is less inclined to the mean plane of atoms C5/O5/C25/O4/C26 [5.67 (4)° compared to 15.50 (2)° in the title compound]. In both structures, a strong intra­molecular hydrogen bond is observed between Bz-group at C5 and the hydroxyl group at C15. However, the presence of three hydroxyl substitutes at C3, C8 and C15 leads to the appearance of four intra­molecular hydrogen bonds in the structure of EZIJAA, which is more loosely packed than that of the title compound and which contains voids.

## Synthesis and crystallization   

The process of extraction and isolation of ES2 is described in detail by Lu *et al.* (2014 ▸). Colourless prismatic single crystals were prepared by slow evaporation of the solvent from an ethanol solution at room temperature. The absolute configuration was been determined as 2*R*,3*R*,4*S*,5*R*,7*S*,8*S*,9*S*,13*S*,14*S*,15*R*, the same as reported by Lu *et al.* (2014 ▸).

## Refinement   

Crystal data, data collection and structure refinement details are summarized in Table 2 ▸. All hydrogen atoms were placed in idealized positions (O—H = 0.82, C—H = 0.93–0.98 Å) and refined as riding atoms. For the hydroxyl group, possible hydrogen-bonding positions were taken into account in generating the idealized position (AFIX 83). *U*_iso_(H) values were set to a multiple of *U*_eq_(C,O) with multipliers of 1.5 for CH_3_ and OH, and 1.2 for CH and CH_2_ units, respectively.

A large difference peak and Hirshfeld test deviations indicated disorder of the C2-acet­oxy group. The disordered atoms were modelled over two positions using the PART instruction with occupancies for the dominant and minor positions of 83% and 17%, respectively. A bond distance restraint to a target value of 1.4 (1) Å was used in the disordered acetyl group (C21*B*–C22*B*).

## Supplementary Material

Crystal structure: contains datablock(s) I. DOI: 10.1107/S205698901901541X/fy2141sup1.cif

Structure factors: contains datablock(s) I. DOI: 10.1107/S205698901901541X/fy2141Isup3.hkl

CCDC reference: 1965699

Additional supporting information:  crystallographic information; 3D view; checkCIF report

## Figures and Tables

**Table 1:** Hydrogen-bond geometry (Å, °)

*D*—H⋯*A*	*D*—H	H⋯*A*	*D*⋯*A*	*D*—H⋯*A*
O3—H3*A*⋯O5	0.82	2.11	2.821 (4)	145
C1—H1*A*⋯O15*B*	0.97	2.25	2.78 (2)	113
C3—H3*B*⋯O11	0.98	2.47	3.330 (6)	147
C4—H4*A*⋯O15*B*	0.98	2.54	3.12 (2)	118
C16—H16*A*⋯O3	0.96	2.62	3.222 (6)	121
C16—H16*C*⋯O15*A*	0.96	2.39	2.946 (7)	116
C37—H37*B*⋯O9^i^	0.96	2.39	3.239 (8)	147
C37—H37*C*⋯O15*B* ^ii^	0.96	2.19	2.92 (2)	132

Symmetry codes: (i) 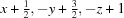; (ii) 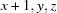.

**Table 2:** Experimental details

Crystal data	
Chemical formula	C_46_H_56_O_15_
*M*_r_	848.90
Crystal system, space group	Orthorhombic, *P*2_1_2_1_2_1_
Temperature (K)	104
*a*, *b*, *c* (Å)	8.9730 (5), 20.9171 (9), 23.9201 (11)
*V* (Å^3^)	4489.5 (4)
*Z*	4
Radiation type	Cu *K*α
μ (mm^−1^)	0.78
Crystal size (mm)	0.3 × 0.2 × 0.1

Data collection	
Diffractometer	Agilent Xcalibur Ruby
Absorption correction	Multi-scan (*CrysAlis PRO*; Agilent, 2014 ▸)
*T*_min_, *T*_max_	0.748, 1.000
No. of measured, independent and observed [*I* > 2σ(*I*)] reflections	37870, 9301, 7007
*R*_int_	0.058
(sin θ/λ)_max_ (Å^−1^)	0.631

Refinement	
*R*[*F* ^2^ > 2σ(*F* ^2^)], *wR*(*F* ^2^), *S*	0.056, 0.153, 1.02
No. of reflections	9301
No. of parameters	587
No. of restraints	1
H-atom treatment	H-atom parameters constrained
Δρ_max_, Δρ_min_ (e Å^−3^)	0.26, −0.19
Absolute structure	Flack *x* determined using 2403 quotients [(*I* ^+^)−(*I* ^−^)]/[(*I* ^+^)+(*I* ^−^)] (Parsons *et al.*, 2013 ▸)
Absolute structure parameter	−0.06 (11)

Computer programs: *CrysAlis PRO* (Agilent, 2014 ▸), *SHELXS97* (Sheldrick, 2008 ▸), *SHELXL2018* (Sheldrick, 2015 ▸) and *XP* (Siemens, 1994 ▸).

## supplementary crystallographic information

### Crystal data

**Table:**

C_46_H_56_O_15_	*D*_x_ = 1.256 Mg m^−^^3^*D*_m_ = 1.256 Mg m^−^^3^*D*_m_ measured by not measured
*M**_r_* = 848.90	Melting point: 166 K
Orthorhombic, *P*2_1_2_1_2_1_	Cu *K*α radiation, λ = 1.54184 Å
*a* = 8.9730 (5) Å	Cell parameters from 5355 reflections
*b* = 20.9171 (9) Å	θ = 4.2–69.6°
*c* = 23.9201 (11) Å	µ = 0.78 mm^−^^1^
*V* = 4489.5 (4) Å^3^	*T* = 104 K
*Z* = 4	Prism, colourless
*F*(000) = 1808	0.3 × 0.2 × 0.1 mm

### Data collection

**Table:**

Agilent Xcalibur Ruby diffractometer	9301 independent reflections
Radiation source: fine-focus sealed tube	7007 reflections with *I* > 2σ(*I*)
Detector resolution: 10.2576 pixels mm^-1^	*R*_int_ = 0.058
ω scans	θ_max_ = 76.5°, θ_min_ = 3.7°
Absorption correction: multi-scan (CrysAlis PRO; Agilent, 2014)	*h* = −11→10
*T*_min_ = 0.748, *T*_max_ = 1.000	*k* = −26→26
37870 measured reflections	*l* = −30→27

### Refinement

**Table:**

Refinement on *F*^2^	Secondary atom site location: difference Fourier map
Least-squares matrix: full	Hydrogen site location: inferred from neighbouring sites
*R*[*F*^2^ > 2σ(*F*^2^)] = 0.056	H-atom parameters constrained
*wR*(*F*^2^) = 0.153	*w* = 1/[σ^2^(*F*_o_^2^) + (0.071*P*)^2^ + 1.4583*P*] where *P* = (*F*_o_^2^ + 2*F*_c_^2^)/3
*S* = 1.02	(Δ/σ)_max_ = 0.010
9301 reflections	Δρ_max_ = 0.26 e Å^−^^3^
587 parameters	Δρ_min_ = −0.19 e Å^−^^3^
1 restraint	Absolute structure: Flack *x* determined using 2403 quotients [(*I*^+^)-(*I*^-^)]/[(*I*^+^)+(*I*^-^)] (Parsons *et al.*, 2013)
Primary atom site location: structure-invariant direct methods	Absolute structure parameter: −0.06 (11)

### Special details

**Table:**

Geometry. All esds (except the esd in the dihedral angle between two l.s. planes) are estimated using the full covariance matrix. The cell esds are taken into account individually in the estimation of esds in distances, angles and torsion angles; correlations between esds in cell parameters are only used when they are defined by crystal symmetry. An approximate (isotropic) treatment of cell esds is used for estimating esds involving l.s. planes.
Refinement. Refinement of F^2^ against ALL reflections. The weighted R-factor wR and goodness of fit S are based on F^2^, conventional R-factors R are based on F, with F set to zero for negative F^2^. The threshold expression of F^2^ > 2sigma(F^2^) is used only for calculating R-factors(gt) etc. and is not relevant to the choice of reflections for refinement. R-factors based on F^2^ are statistically about twice as large as those based on F, and R- factors based on ALL data will be even larger.

### Fractional atomic coordinates and isotropic or equivalent isotropic displacement parameters (Å^2^)

**Table:**

	*x*	*y*	*z*	*U*_iso_*/*U*_eq_	Occ. (<1)
O1	0.0006 (4)	0.69300 (16)	0.89766 (13)	0.0618 (9)	
O2	0.1295 (3)	0.67482 (14)	0.81877 (12)	0.0470 (7)	
O3	0.0829 (3)	0.54758 (14)	0.83474 (12)	0.0480 (7)	
H3A	0.173963	0.544145	0.834484	0.072*	
O4	0.5306 (4)	0.64154 (17)	0.78021 (13)	0.0559 (8)	
O5	0.3694 (3)	0.55943 (14)	0.78729 (12)	0.0444 (6)	
O6	0.5606 (3)	0.62727 (17)	0.58845 (13)	0.0546 (8)	
O7	0.7062 (4)	0.6966 (2)	0.63330 (16)	0.0720 (11)	
O8	0.2610 (4)	0.67436 (14)	0.54698 (12)	0.0483 (7)	
O9	0.3759 (5)	0.7351 (2)	0.48129 (16)	0.0840 (13)	
O10	0.3579 (4)	0.54361 (15)	0.58592 (12)	0.0503 (7)	
O11	0.1132 (4)	0.52049 (15)	0.58730 (15)	0.0577 (8)	
O12	0.2136 (3)	0.44549 (13)	0.75343 (12)	0.0473 (7)	
O13	0.2862 (5)	0.3971 (2)	0.67361 (18)	0.0914 (15)	
C1	−0.1069 (5)	0.5379 (2)	0.7667 (2)	0.0487 (11)	
H1A	−0.162596	0.561225	0.738581	0.058*	
H1B	−0.170118	0.531620	0.799144	0.058*	
C2	−0.0527 (5)	0.47277 (19)	0.7433 (2)	0.0487 (11)	
C3	0.1086 (5)	0.4847 (2)	0.72294 (18)	0.0440 (9)	
H3B	0.116083	0.475813	0.682811	0.053*	
C4	0.1368 (5)	0.55634 (19)	0.73401 (17)	0.0411 (9)	
H4A	0.093509	0.578117	0.701564	0.049*	
C5	0.2995 (5)	0.5796 (2)	0.73579 (17)	0.0426 (9)	
H5A	0.298412	0.626464	0.735278	0.051*	
C6	0.3880 (5)	0.5570 (2)	0.68555 (18)	0.0480 (10)	
C7	0.3389 (5)	0.5878 (2)	0.63125 (17)	0.0439 (10)	
H7A	0.233527	0.599777	0.634048	0.053*	
C8	0.4323 (5)	0.6479 (2)	0.62011 (18)	0.0479 (10)	
H8A	0.467627	0.664157	0.656185	0.057*	
C9	0.3534 (5)	0.7022 (2)	0.59007 (18)	0.0479 (10)	
H9A	0.429542	0.728441	0.571698	0.058*	
C10	0.2576 (5)	0.7471 (2)	0.62756 (18)	0.0479 (10)	
C11	0.1315 (5)	0.7128 (2)	0.65684 (18)	0.0463 (10)	
H11A	0.069291	0.687982	0.634587	0.056*	
C12	0.1009 (5)	0.7145 (2)	0.71069 (18)	0.0444 (10)	
H12A	0.169386	0.735170	0.733586	0.053*	
C13	−0.0346 (5)	0.68617 (19)	0.73900 (19)	0.0450 (10)	
H13A	−0.085897	0.658635	0.712004	0.054*	
C14	0.0022 (5)	0.64604 (19)	0.79088 (18)	0.0447 (10)	
H14A	−0.082849	0.649530	0.816401	0.054*	
C15	0.0340 (5)	0.5742 (2)	0.78276 (18)	0.0432 (9)	
C16	−0.0717 (6)	0.4202 (2)	0.7860 (2)	0.0641 (14)	
H16A	−0.018193	0.431146	0.819420	0.096*	
H16B	−0.175563	0.415222	0.794593	0.096*	
H16C	−0.033412	0.380874	0.771149	0.096*	
C17	0.5020 (6)	0.5180 (3)	0.6878 (2)	0.0649 (14)	
H17A	0.555265	0.508527	0.655517	0.097*	
H17B	0.529653	0.499790	0.721730	0.097*	
C18	0.3639 (6)	0.7802 (2)	0.6688 (2)	0.0549 (12)	
H18A	0.441873	0.801291	0.648462	0.082*	
H18B	0.309531	0.811149	0.690370	0.082*	
H18C	0.406548	0.748918	0.693431	0.082*	
C19	0.1882 (6)	0.7986 (2)	0.5893 (2)	0.0585 (12)	
H19A	0.266006	0.821231	0.570141	0.088*	
H19B	0.123781	0.778591	0.562493	0.088*	
H19C	0.131616	0.828133	0.611582	0.088*	
C20	−0.1399 (6)	0.7408 (2)	0.7548 (2)	0.0549 (11)	
H20A	−0.226740	0.723714	0.772888	0.082*	
H20B	−0.089849	0.769599	0.779842	0.082*	
H20C	−0.169221	0.763488	0.721702	0.082*	
C23	0.2943 (6)	0.4030 (3)	0.7229 (2)	0.0626 (14)	
C24	0.4005 (6)	0.3686 (3)	0.7603 (3)	0.0712 (16)	
H24A	0.384613	0.381844	0.798304	0.107*	
H24B	0.384338	0.323385	0.757292	0.107*	
H24C	0.500928	0.378485	0.749489	0.107*	
C25	0.4866 (5)	0.5943 (2)	0.80471 (18)	0.0457 (10)	
C26	0.5574 (5)	0.5679 (2)	0.85550 (18)	0.0469 (10)	
C27	0.5303 (6)	0.5046 (2)	0.8724 (2)	0.0548 (12)	
H27A	0.460643	0.479562	0.853638	0.066*	
C28	0.6074 (7)	0.4804 (3)	0.9166 (2)	0.0652 (14)	
H28A	0.589648	0.438648	0.928197	0.078*	
C29	0.7118 (7)	0.5171 (3)	0.9445 (2)	0.0659 (14)	
H29A	0.765091	0.499456	0.974015	0.079*	
C30	0.7379 (6)	0.5799 (3)	0.9288 (2)	0.0580 (12)	
H30A	0.805632	0.605031	0.948348	0.070*	
C31	0.6621 (5)	0.6040 (2)	0.88420 (19)	0.0503 (11)	
H31A	0.681026	0.645716	0.872697	0.060*	
C32	0.2368 (6)	0.5102 (2)	0.5696 (2)	0.0524 (11)	
C33	0.2844 (8)	0.4574 (3)	0.5295 (3)	0.0758 (17)	
H33A	0.364841	0.433760	0.548003	0.091*	
C34	0.1575 (8)	0.4108 (3)	0.5216 (3)	0.097 (2)	
H34A	0.123433	0.396131	0.557373	0.145*	
H34B	0.077123	0.431684	0.502355	0.145*	
H34C	0.191245	0.375006	0.499867	0.145*	
C35	0.3466 (11)	0.4834 (4)	0.4777 (3)	0.125 (3)	
H35A	0.426177	0.512376	0.486541	0.187*	
H35B	0.384281	0.449103	0.455127	0.187*	
H35C	0.270159	0.505780	0.457616	0.187*	
C36	0.6929 (5)	0.6534 (3)	0.60053 (19)	0.0543 (12)	
C37	0.8145 (6)	0.6231 (3)	0.5679 (2)	0.0679 (14)	
H37A	0.774040	0.589746	0.544923	0.102*	
H37B	0.861401	0.654651	0.544694	0.102*	
H37C	0.886841	0.605300	0.593087	0.102*	
C38	0.2832 (6)	0.6946 (3)	0.4941 (2)	0.0600 (13)	
C39	0.1787 (8)	0.6624 (3)	0.4550 (2)	0.0738 (17)	
H39A	0.117637	0.632800	0.475338	0.111*	
H39B	0.116467	0.693873	0.437353	0.111*	
H39C	0.234405	0.639867	0.426981	0.111*	
C40	0.1127 (6)	0.69846 (19)	0.87095 (17)	0.0471 (10)	
C41	0.2520 (6)	0.73057 (18)	0.89048 (17)	0.0463 (11)	
C42	0.3702 (6)	0.7442 (2)	0.85517 (19)	0.0537 (12)	
H42A	0.364942	0.733140	0.817572	0.064*	
C43	0.4957 (6)	0.7742 (2)	0.8757 (2)	0.0589 (13)	
H43A	0.574525	0.783603	0.851844	0.071*	
C44	0.5043 (7)	0.7901 (2)	0.9311 (2)	0.0615 (14)	
H44A	0.589471	0.810125	0.944670	0.074*	
C45	0.3878 (7)	0.7768 (2)	0.9670 (2)	0.0650 (15)	
H45A	0.394730	0.787287	1.004668	0.078*	
C46	0.2621 (7)	0.7480 (2)	0.94659 (19)	0.0556 (12)	
H46A	0.182458	0.740016	0.970464	0.067*	
O14A	−0.1487 (7)	0.4643 (3)	0.6922 (3)	0.0503 (15)	0.826 (8)
O15A	−0.0385 (5)	0.37019 (18)	0.6716 (2)	0.0693 (15)	0.826 (8)
C21A	−0.1251 (7)	0.4117 (3)	0.6604 (3)	0.0609 (18)	0.826 (8)
C22A	−0.2263 (13)	0.4133 (4)	0.6105 (5)	0.077 (3)	0.826 (8)
H22A	−0.284604	0.451803	0.611333	0.116*	0.826 (8)
H22B	−0.167759	0.412314	0.576958	0.116*	0.826 (8)
H22C	−0.291418	0.376913	0.611383	0.116*	0.826 (8)
O15B	−0.122 (2)	0.5249 (12)	0.6513 (8)	0.062 (6)	0.174 (8)
O14B	−0.139 (3)	0.4383 (12)	0.7081 (13)	0.050 (7)	0.174 (8)
C21B	−0.160 (3)	0.4701 (18)	0.6577 (16)	0.054 (8)	0.174 (8)
C22B	−0.207 (5)	0.434 (3)	0.611 (2)	0.10 (2)	0.174 (8)
H22D	−0.232776	0.391506	0.623405	0.151*	0.174 (8)
H22E	−0.292928	0.453841	0.594859	0.151*	0.174 (8)
H22F	−0.128428	0.431672	0.584298	0.151*	0.174 (8)

### Atomic displacement parameters (Å^2^)

**Table:**

	*U*^11^	*U*^22^	*U*^33^	*U*^12^	*U*^13^	*U*^23^
O1	0.071 (2)	0.061 (2)	0.0533 (19)	−0.0157 (18)	0.0232 (18)	−0.0141 (15)
O2	0.0501 (17)	0.0486 (15)	0.0422 (15)	−0.0141 (14)	0.0098 (14)	−0.0116 (13)
O3	0.0454 (17)	0.0487 (16)	0.0499 (17)	−0.0067 (13)	0.0091 (14)	−0.0044 (13)
O4	0.0495 (19)	0.071 (2)	0.0467 (17)	−0.0214 (17)	0.0017 (15)	0.0036 (15)
O5	0.0385 (15)	0.0516 (16)	0.0431 (15)	−0.0053 (13)	−0.0045 (13)	−0.0031 (13)
O6	0.0378 (16)	0.080 (2)	0.0455 (17)	−0.0068 (16)	0.0021 (14)	−0.0171 (16)
O7	0.053 (2)	0.096 (3)	0.067 (2)	−0.021 (2)	0.0049 (18)	−0.023 (2)
O8	0.0508 (18)	0.0529 (16)	0.0410 (15)	−0.0042 (14)	−0.0002 (14)	−0.0088 (13)
O9	0.078 (3)	0.118 (3)	0.056 (2)	−0.026 (3)	0.012 (2)	0.000 (2)
O10	0.0503 (18)	0.0554 (17)	0.0452 (16)	−0.0009 (15)	0.0044 (15)	−0.0169 (13)
O11	0.051 (2)	0.0502 (18)	0.072 (2)	−0.0022 (15)	−0.0154 (17)	−0.0163 (16)
O12	0.0456 (17)	0.0435 (15)	0.0529 (17)	0.0066 (13)	−0.0095 (14)	−0.0092 (13)
O13	0.092 (3)	0.107 (3)	0.075 (3)	0.062 (3)	−0.033 (2)	−0.044 (2)
C1	0.043 (2)	0.039 (2)	0.064 (3)	−0.0049 (18)	0.005 (2)	−0.0133 (19)
C2	0.041 (2)	0.036 (2)	0.069 (3)	−0.0022 (17)	−0.005 (2)	−0.014 (2)
C3	0.039 (2)	0.044 (2)	0.049 (2)	0.0061 (18)	−0.0042 (19)	−0.0043 (18)
C4	0.039 (2)	0.041 (2)	0.044 (2)	−0.0029 (18)	0.0007 (18)	−0.0091 (17)
C5	0.037 (2)	0.051 (2)	0.040 (2)	−0.0010 (18)	0.0013 (17)	−0.0046 (17)
C6	0.034 (2)	0.061 (3)	0.049 (2)	0.001 (2)	−0.0008 (19)	−0.007 (2)
C7	0.039 (2)	0.053 (2)	0.040 (2)	−0.0027 (19)	0.0045 (18)	−0.0150 (18)
C8	0.042 (2)	0.062 (3)	0.039 (2)	−0.008 (2)	0.0038 (19)	−0.017 (2)
C9	0.044 (2)	0.057 (2)	0.042 (2)	−0.013 (2)	0.0043 (19)	−0.0109 (19)
C10	0.049 (3)	0.049 (2)	0.046 (2)	−0.008 (2)	0.006 (2)	−0.0117 (19)
C11	0.044 (2)	0.043 (2)	0.052 (2)	−0.0085 (19)	0.001 (2)	−0.0147 (18)
C12	0.042 (2)	0.044 (2)	0.048 (2)	−0.0032 (18)	0.001 (2)	−0.0103 (18)
C13	0.039 (2)	0.041 (2)	0.055 (2)	−0.0051 (18)	0.0085 (19)	−0.0129 (18)
C14	0.041 (2)	0.045 (2)	0.048 (2)	−0.0096 (18)	0.012 (2)	−0.0153 (18)
C15	0.039 (2)	0.045 (2)	0.046 (2)	−0.0057 (18)	0.0046 (18)	−0.0057 (18)
C16	0.059 (3)	0.044 (2)	0.089 (4)	−0.004 (2)	0.013 (3)	−0.002 (2)
C17	0.048 (3)	0.088 (4)	0.059 (3)	0.013 (3)	0.009 (2)	−0.007 (3)
C18	0.052 (3)	0.057 (3)	0.056 (3)	−0.020 (2)	0.009 (2)	−0.018 (2)
C19	0.063 (3)	0.052 (2)	0.060 (3)	−0.008 (2)	0.012 (2)	−0.010 (2)
C20	0.050 (3)	0.050 (2)	0.065 (3)	−0.002 (2)	0.013 (2)	−0.010 (2)
C23	0.052 (3)	0.068 (3)	0.068 (3)	0.016 (2)	−0.022 (2)	−0.019 (3)
C24	0.060 (3)	0.064 (3)	0.090 (4)	0.021 (3)	−0.030 (3)	−0.015 (3)
C25	0.034 (2)	0.063 (3)	0.040 (2)	−0.008 (2)	0.0044 (17)	−0.0092 (19)
C26	0.038 (2)	0.055 (3)	0.047 (2)	−0.0015 (19)	0.0010 (19)	−0.0046 (19)
C27	0.049 (3)	0.064 (3)	0.052 (3)	−0.008 (2)	−0.003 (2)	−0.008 (2)
C28	0.067 (4)	0.058 (3)	0.071 (3)	−0.004 (3)	−0.012 (3)	0.003 (2)
C29	0.060 (3)	0.080 (3)	0.057 (3)	−0.003 (3)	−0.015 (3)	0.005 (3)
C30	0.050 (3)	0.072 (3)	0.052 (3)	−0.013 (3)	−0.008 (2)	−0.003 (2)
C31	0.045 (3)	0.060 (3)	0.045 (2)	−0.010 (2)	0.005 (2)	−0.005 (2)
C32	0.056 (3)	0.049 (2)	0.052 (3)	0.005 (2)	−0.008 (2)	−0.013 (2)
C33	0.093 (5)	0.061 (3)	0.073 (4)	0.012 (3)	−0.016 (3)	−0.028 (3)
C34	0.101 (5)	0.067 (4)	0.122 (6)	0.008 (4)	−0.030 (5)	−0.046 (4)
C35	0.142 (8)	0.132 (7)	0.100 (6)	0.007 (6)	0.024 (6)	−0.069 (5)
C36	0.042 (3)	0.077 (3)	0.044 (2)	−0.009 (2)	−0.004 (2)	−0.003 (2)
C37	0.045 (3)	0.090 (4)	0.069 (3)	−0.011 (3)	0.007 (3)	−0.009 (3)
C38	0.059 (3)	0.075 (3)	0.045 (3)	0.005 (3)	0.003 (2)	−0.010 (2)
C39	0.092 (5)	0.074 (3)	0.056 (3)	0.010 (3)	−0.025 (3)	−0.010 (3)
C40	0.067 (3)	0.038 (2)	0.037 (2)	−0.008 (2)	0.009 (2)	−0.0032 (16)
C41	0.071 (3)	0.0301 (18)	0.038 (2)	−0.006 (2)	−0.005 (2)	0.0001 (16)
C42	0.069 (3)	0.051 (2)	0.041 (2)	−0.015 (2)	−0.004 (2)	−0.0003 (19)
C43	0.070 (3)	0.054 (3)	0.053 (3)	−0.020 (2)	−0.015 (3)	0.007 (2)
C44	0.079 (4)	0.039 (2)	0.066 (3)	−0.014 (2)	−0.029 (3)	0.005 (2)
C45	0.099 (5)	0.053 (3)	0.043 (3)	−0.001 (3)	−0.023 (3)	−0.007 (2)
C46	0.080 (4)	0.043 (2)	0.043 (2)	−0.002 (2)	−0.001 (2)	−0.0023 (19)
O14A	0.043 (3)	0.031 (3)	0.077 (5)	0.004 (2)	−0.021 (3)	−0.013 (3)
O15A	0.077 (3)	0.039 (2)	0.092 (3)	0.019 (2)	−0.028 (3)	−0.021 (2)
C21A	0.055 (4)	0.037 (3)	0.091 (5)	0.000 (3)	−0.021 (4)	−0.018 (3)
C22A	0.085 (7)	0.040 (4)	0.107 (6)	0.001 (4)	−0.052 (5)	−0.025 (4)
O15B	0.047 (11)	0.087 (17)	0.053 (12)	0.007 (11)	−0.011 (9)	0.003 (10)
O14B	0.036 (11)	0.044 (15)	0.070 (19)	0.005 (12)	0.003 (11)	−0.019 (12)
C21B	0.032 (14)	0.07 (2)	0.06 (2)	0.005 (13)	−0.025 (15)	0.003 (18)
C22B	0.040 (19)	0.09 (4)	0.17 (5)	−0.03 (2)	0.03 (3)	−0.10 (4)

### Geometric parameters (Å, º)

**Table:**

O1—C40	1.197 (5)	C19—H19B	0.9600
O2—C40	1.351 (5)	C19—H19C	0.9600
O2—C14	1.454 (5)	C20—H20A	0.9600
O3—C15	1.432 (5)	C20—H20B	0.9600
O3—H3A	0.8200	C20—H20C	0.9600
O4—C25	1.214 (5)	C23—C24	1.492 (7)
O5—C25	1.347 (5)	C24—H24A	0.9600
O5—C5	1.445 (5)	C24—H24B	0.9600
O6—C36	1.339 (6)	C24—H24C	0.9600
O6—C8	1.444 (5)	C25—C26	1.478 (6)
O7—C36	1.203 (6)	C26—C31	1.388 (6)
O8—C38	1.349 (6)	C26—C27	1.406 (7)
O8—C9	1.446 (5)	C27—C28	1.361 (7)
O9—C38	1.225 (7)	C27—H27A	0.9300
O10—C32	1.350 (6)	C28—C29	1.382 (8)
O10—C7	1.435 (5)	C28—H28A	0.9300
O11—C32	1.206 (6)	C29—C30	1.386 (8)
O12—C23	1.359 (6)	C29—H29A	0.9300
O12—C3	1.447 (5)	C30—C31	1.362 (7)
O13—C23	1.188 (7)	C30—H30A	0.9300
C1—C15	1.523 (6)	C31—H31A	0.9300
C1—C2	1.552 (6)	C32—C33	1.525 (7)
C1—H1A	0.9700	C33—C35	1.464 (10)
C1—H1B	0.9700	C33—C34	1.510 (9)
C2—O14B	1.35 (3)	C33—H33A	0.9800
C2—O14A	1.505 (8)	C34—H34A	0.9600
C2—C16	1.510 (7)	C34—H34B	0.9600
C2—C3	1.548 (6)	C34—H34C	0.9600
C3—C4	1.542 (6)	C35—H35A	0.9600
C3—H3B	0.9800	C35—H35B	0.9600
C4—C15	1.533 (6)	C35—H35C	0.9600
C4—C5	1.539 (6)	C36—C37	1.483 (7)
C4—H4A	0.9800	C37—H37A	0.9600
C5—C6	1.516 (6)	C37—H37B	0.9600
C5—H5A	0.9800	C37—H37C	0.9600
C6—C17	1.309 (7)	C38—C39	1.486 (8)
C6—C7	1.516 (6)	C39—H39A	0.9600
C7—C8	1.534 (6)	C39—H39B	0.9600
C7—H7A	0.9800	C39—H39C	0.9600
C8—C9	1.519 (7)	C40—C41	1.494 (7)
C8—H8A	0.9800	C41—C42	1.386 (7)
C9—C10	1.557 (6)	C41—C46	1.394 (6)
C9—H9A	0.9800	C42—C43	1.379 (7)
C10—C11	1.511 (6)	C42—H42A	0.9300
C10—C18	1.538 (6)	C43—C44	1.368 (7)
C10—C19	1.545 (7)	C43—H43A	0.9300
C11—C12	1.318 (6)	C44—C45	1.382 (8)
C11—H11A	0.9300	C44—H44A	0.9300
C12—C13	1.513 (6)	C45—C46	1.368 (8)
C12—H12A	0.9300	C45—H45A	0.9300
C13—C20	1.531 (6)	C46—H46A	0.9300
C13—C14	1.534 (6)	O14A—C21A	1.355 (9)
C13—H13A	0.9800	O15A—C21A	1.195 (7)
C14—C15	1.541 (6)	C21A—C22A	1.499 (11)
C14—H14A	0.9800	C22A—H22A	0.9600
C16—H16A	0.9600	C22A—H22B	0.9600
C16—H16B	0.9600	C22A—H22C	0.9600
C16—H16C	0.9600	O15B—C21B	1.20 (4)
C17—H17A	0.9300	O14B—C21B	1.39 (5)
C17—H17B	0.9300	C21B—C22B	1.407 (14)
C18—H18A	0.9600	C22B—H22D	0.9600
C18—H18B	0.9600	C22B—H22E	0.9600
C18—H18C	0.9600	C22B—H22F	0.9600
C19—H19A	0.9600		
			
C40—O2—C14	119.2 (3)	H20A—C20—H20B	109.5
C15—O3—H3A	109.5	C13—C20—H20C	109.5
C25—O5—C5	116.4 (3)	H20A—C20—H20C	109.5
C36—O6—C8	118.2 (3)	H20B—C20—H20C	109.5
C38—O8—C9	117.2 (4)	O13—C23—O12	124.7 (5)
C32—O10—C7	117.1 (3)	O13—C23—C24	125.7 (5)
C23—O12—C3	116.6 (3)	O12—C23—C24	109.5 (4)
C15—C1—C2	105.6 (4)	C23—C24—H24A	109.5
C15—C1—H1A	110.6	C23—C24—H24B	109.5
C2—C1—H1A	110.6	H24A—C24—H24B	109.5
C15—C1—H1B	110.6	C23—C24—H24C	109.5
C2—C1—H1B	110.6	H24A—C24—H24C	109.5
H1A—C1—H1B	108.7	H24B—C24—H24C	109.5
O14B—C2—C16	88.2 (13)	O4—C25—O5	123.1 (4)
O14A—C2—C16	113.5 (4)	O4—C25—C26	124.1 (4)
O14B—C2—C3	115.2 (12)	O5—C25—C26	112.8 (4)
O14A—C2—C3	107.4 (4)	C31—C26—C27	119.2 (4)
C16—C2—C3	115.8 (4)	C31—C26—C25	119.6 (4)
O14B—C2—C1	120.9 (11)	C27—C26—C25	121.0 (4)
O14A—C2—C1	102.6 (4)	C28—C27—C26	119.0 (5)
C16—C2—C1	111.1 (4)	C28—C27—H27A	120.5
C3—C2—C1	105.3 (3)	C26—C27—H27A	120.5
O12—C3—C4	111.0 (3)	C27—C28—C29	120.9 (5)
O12—C3—C2	111.0 (4)	C27—C28—H28A	119.6
C4—C3—C2	104.8 (3)	C29—C28—H28A	119.6
O12—C3—H3B	110.0	C28—C29—C30	120.7 (5)
C4—C3—H3B	110.0	C28—C29—H29A	119.6
C2—C3—H3B	110.0	C30—C29—H29A	119.7
C15—C4—C5	118.2 (3)	C31—C30—C29	118.6 (5)
C15—C4—C3	105.6 (3)	C31—C30—H30A	120.7
C5—C4—C3	117.9 (4)	C29—C30—H30A	120.7
C15—C4—H4A	104.5	C30—C31—C26	121.6 (5)
C5—C4—H4A	104.5	C30—C31—H31A	119.2
C3—C4—H4A	104.5	C26—C31—H31A	119.2
O5—C5—C6	110.9 (3)	O11—C32—O10	123.2 (4)
O5—C5—C4	110.0 (3)	O11—C32—C33	127.4 (5)
C6—C5—C4	112.1 (3)	O10—C32—C33	109.4 (5)
O5—C5—H5A	107.9	C35—C33—C34	114.9 (6)
C6—C5—H5A	107.9	C35—C33—C32	111.7 (5)
C4—C5—H5A	107.9	C34—C33—C32	109.6 (6)
C17—C6—C7	121.9 (4)	C35—C33—H33A	106.7
C17—C6—C5	124.8 (5)	C34—C33—H33A	106.7
C7—C6—C5	113.2 (4)	C32—C33—H33A	106.7
O10—C7—C6	109.8 (4)	C33—C34—H34A	109.5
O10—C7—C8	109.4 (3)	C33—C34—H34B	109.5
C6—C7—C8	109.8 (4)	H34A—C34—H34B	109.5
O10—C7—H7A	109.3	C33—C34—H34C	109.5
C6—C7—H7A	109.3	H34A—C34—H34C	109.5
C8—C7—H7A	109.3	H34B—C34—H34C	109.5
O6—C8—C9	110.3 (4)	C33—C35—H35A	109.5
O6—C8—C7	106.4 (3)	C33—C35—H35B	109.5
C9—C8—C7	116.1 (4)	H35A—C35—H35B	109.5
O6—C8—H8A	107.9	C33—C35—H35C	109.5
C9—C8—H8A	107.9	H35A—C35—H35C	109.5
C7—C8—H8A	107.9	H35B—C35—H35C	109.5
O8—C9—C8	107.7 (3)	O7—C36—O6	122.4 (5)
O8—C9—C10	109.7 (4)	O7—C36—C37	126.2 (5)
C8—C9—C10	115.8 (4)	O6—C36—C37	111.4 (4)
O8—C9—H9A	107.8	C36—C37—H37A	109.5
C8—C9—H9A	107.8	C36—C37—H37B	109.5
C10—C9—H9A	107.8	H37A—C37—H37B	109.5
C11—C10—C18	112.4 (4)	C36—C37—H37C	109.5
C11—C10—C19	107.7 (4)	H37A—C37—H37C	109.5
C18—C10—C19	108.4 (4)	H37B—C37—H37C	109.5
C11—C10—C9	113.2 (4)	O9—C38—O8	123.5 (5)
C18—C10—C9	107.4 (4)	O9—C38—C39	125.7 (5)
C19—C10—C9	107.6 (4)	O8—C38—C39	110.8 (5)
C12—C11—C10	126.6 (4)	C38—C39—H39A	109.5
C12—C11—H11A	116.7	C38—C39—H39B	109.5
C10—C11—H11A	116.7	H39A—C39—H39B	109.5
C11—C12—C13	126.5 (4)	C38—C39—H39C	109.5
C11—C12—H12A	116.7	H39A—C39—H39C	109.5
C13—C12—H12A	116.7	H39B—C39—H39C	109.5
C12—C13—C20	108.3 (3)	O1—C40—O2	123.5 (4)
C12—C13—C14	113.8 (4)	O1—C40—C41	125.4 (4)
C20—C13—C14	110.0 (4)	O2—C40—C41	111.1 (4)
C12—C13—H13A	108.2	C42—C41—C46	118.9 (5)
C20—C13—H13A	108.2	C42—C41—C40	122.9 (4)
C14—C13—H13A	108.2	C46—C41—C40	118.2 (4)
O2—C14—C13	108.3 (3)	C43—C42—C41	120.1 (4)
O2—C14—C15	108.4 (4)	C43—C42—H42A	119.9
C13—C14—C15	118.1 (3)	C41—C42—H42A	119.9
O2—C14—H14A	107.2	C44—C43—C42	120.1 (5)
C13—C14—H14A	107.2	C44—C43—H43A	119.9
C15—C14—H14A	107.2	C42—C43—H43A	119.9
O3—C15—C1	106.2 (3)	C43—C44—C45	120.7 (5)
O3—C15—C4	112.4 (4)	C43—C44—H44A	119.7
C1—C15—C4	100.7 (3)	C45—C44—H44A	119.7
O3—C15—C14	109.1 (3)	C46—C45—C44	119.4 (4)
C1—C15—C14	111.3 (4)	C46—C45—H45A	120.3
C4—C15—C14	116.4 (3)	C44—C45—H45A	120.3
C2—C16—H16A	109.5	C45—C46—C41	120.8 (5)
C2—C16—H16B	109.5	C45—C46—H46A	119.6
H16A—C16—H16B	109.5	C41—C46—H46A	119.6
C2—C16—H16C	109.5	C21A—O14A—C2	117.6 (5)
H16A—C16—H16C	109.5	O15A—C21A—O14A	124.4 (6)
H16B—C16—H16C	109.5	O15A—C21A—C22A	126.0 (6)
C6—C17—H17A	120.0	O14A—C21A—C22A	109.5 (6)
C6—C17—H17B	120.0	C21A—C22A—H22A	109.5
H17A—C17—H17B	120.0	C21A—C22A—H22B	109.5
C10—C18—H18A	109.5	H22A—C22A—H22B	109.5
C10—C18—H18B	109.5	C21A—C22A—H22C	109.5
H18A—C18—H18B	109.5	H22A—C22A—H22C	109.5
C10—C18—H18C	109.5	H22B—C22A—H22C	109.5
H18A—C18—H18C	109.5	C2—O14B—C21B	111 (2)
H18B—C18—H18C	109.5	O15B—C21B—O14B	122 (3)
C10—C19—H19A	109.5	O15B—C21B—C22B	120 (4)
C10—C19—H19B	109.5	O14B—C21B—C22B	118 (4)
H19A—C19—H19B	109.5	C21B—C22B—H22D	109.5
C10—C19—H19C	109.5	C21B—C22B—H22E	109.5
H19A—C19—H19C	109.5	H22D—C22B—H22E	109.5
H19B—C19—H19C	109.5	C21B—C22B—H22F	109.5
C13—C20—H20A	109.5	H22D—C22B—H22F	109.5
C13—C20—H20B	109.5	H22E—C22B—H22F	109.5
			
C15—C1—C2—O14B	−156.8 (15)	C2—C1—C15—C4	39.8 (5)
C15—C1—C2—O14A	−136.3 (4)	C2—C1—C15—C14	163.9 (4)
C15—C1—C2—C16	102.2 (5)	C5—C4—C15—O3	−62.9 (5)
C15—C1—C2—C3	−24.0 (5)	C3—C4—C15—O3	71.6 (4)
C23—O12—C3—C4	−125.1 (4)	C5—C4—C15—C1	−175.5 (4)
C23—O12—C3—C2	118.8 (4)	C3—C4—C15—C1	−41.1 (4)
O14B—C2—C3—O12	−106.0 (13)	C5—C4—C15—C14	64.0 (5)
O14A—C2—C3—O12	−133.0 (4)	C3—C4—C15—C14	−161.6 (4)
C16—C2—C3—O12	−5.0 (5)	O2—C14—C15—O3	49.6 (4)
C1—C2—C3—O12	118.2 (4)	C13—C14—C15—O3	173.1 (3)
O14B—C2—C3—C4	134.2 (13)	O2—C14—C15—C1	166.4 (3)
O14A—C2—C3—C4	107.1 (4)	C13—C14—C15—C1	−70.0 (5)
C16—C2—C3—C4	−124.9 (4)	O2—C14—C15—C4	−78.9 (4)
C1—C2—C3—C4	−1.7 (5)	C13—C14—C15—C4	44.7 (5)
O12—C3—C4—C15	−93.2 (4)	C3—O12—C23—O13	1.4 (8)
C2—C3—C4—C15	26.7 (4)	C3—O12—C23—C24	177.8 (4)
O12—C3—C4—C5	41.4 (5)	C5—O5—C25—O4	2.2 (6)
C2—C3—C4—C5	161.3 (4)	C5—O5—C25—C26	−176.2 (3)
C25—O5—C5—C6	79.4 (4)	O4—C25—C26—C31	12.9 (7)
C25—O5—C5—C4	−155.9 (3)	O5—C25—C26—C31	−168.7 (4)
C15—C4—C5—O5	54.8 (5)	O4—C25—C26—C27	−161.3 (5)
C3—C4—C5—O5	−74.2 (5)	O5—C25—C26—C27	17.0 (6)
C15—C4—C5—C6	178.7 (4)	C31—C26—C27—C28	0.1 (7)
C3—C4—C5—C6	49.8 (5)	C25—C26—C27—C28	174.4 (5)
O5—C5—C6—C17	7.8 (6)	C26—C27—C28—C29	−0.4 (8)
C4—C5—C6—C17	−115.7 (5)	C27—C28—C29—C30	1.5 (9)
O5—C5—C6—C7	−168.1 (3)	C28—C29—C30—C31	−2.2 (9)
C4—C5—C6—C7	68.4 (5)	C29—C30—C31—C26	1.9 (8)
C32—O10—C7—C6	97.3 (4)	C27—C26—C31—C30	−0.9 (7)
C32—O10—C7—C8	−142.2 (4)	C25—C26—C31—C30	−175.2 (4)
C17—C6—C7—O10	36.6 (6)	C7—O10—C32—O11	7.8 (7)
C5—C6—C7—O10	−147.4 (4)	C7—O10—C32—C33	−169.2 (4)
C17—C6—C7—C8	−83.7 (5)	O11—C32—C33—C35	118.8 (7)
C5—C6—C7—C8	92.4 (4)	O10—C32—C33—C35	−64.4 (7)
C36—O6—C8—C9	93.3 (5)	O11—C32—C33—C34	−9.7 (8)
C36—O6—C8—C7	−140.1 (4)	O10—C32—C33—C34	167.1 (5)
O10—C7—C8—O6	−30.8 (5)	C8—O6—C36—O7	−6.2 (7)
C6—C7—C8—O6	89.7 (4)	C8—O6—C36—C37	175.0 (4)
O10—C7—C8—C9	92.3 (4)	C9—O8—C38—O9	−0.4 (7)
C6—C7—C8—C9	−147.2 (4)	C9—O8—C38—C39	−179.1 (4)
C38—O8—C9—C8	−123.8 (4)	C14—O2—C40—O1	5.7 (7)
C38—O8—C9—C10	109.4 (4)	C14—O2—C40—C41	−175.4 (3)
O6—C8—C9—O8	80.8 (4)	O1—C40—C41—C42	−169.8 (5)
C7—C8—C9—O8	−40.2 (5)	O2—C40—C41—C42	11.3 (6)
O6—C8—C9—C10	−156.1 (4)	O1—C40—C41—C46	9.5 (7)
C7—C8—C9—C10	82.9 (4)	O2—C40—C41—C46	−169.4 (4)
O8—C9—C10—C11	60.7 (5)	C46—C41—C42—C43	0.4 (7)
C8—C9—C10—C11	−61.3 (5)	C40—C41—C42—C43	179.7 (4)
O8—C9—C10—C18	−174.6 (4)	C41—C42—C43—C44	0.5 (8)
C8—C9—C10—C18	63.3 (5)	C42—C43—C44—C45	−0.4 (8)
O8—C9—C10—C19	−58.2 (5)	C43—C44—C45—C46	−0.7 (8)
C8—C9—C10—C19	179.8 (4)	C44—C45—C46—C41	1.7 (7)
C18—C10—C11—C12	6.6 (7)	C42—C41—C46—C45	−1.5 (7)
C19—C10—C11—C12	−112.6 (5)	C40—C41—C46—C45	179.1 (4)
C9—C10—C11—C12	128.5 (5)	C16—C2—O14A—C21A	−62.3 (7)
C10—C11—C12—C13	172.8 (4)	C3—C2—O14A—C21A	67.0 (7)
C11—C12—C13—C20	−105.5 (5)	C1—C2—O14A—C21A	177.7 (6)
C11—C12—C13—C14	131.9 (5)	C2—O14A—C21A—O15A	3.8 (11)
C40—O2—C14—C13	116.5 (4)	C2—O14A—C21A—C22A	−177.6 (7)
C40—O2—C14—C15	−114.2 (4)	C16—C2—O14B—C21B	179 (2)
C12—C13—C14—O2	34.6 (4)	C3—C2—O14B—C21B	−63 (2)
C20—C13—C14—O2	−87.1 (4)	C1—C2—O14B—C21B	65 (3)
C12—C13—C14—C15	−89.0 (4)	C2—O14B—C21B—O15B	−10 (4)
C20—C13—C14—C15	149.3 (4)	C2—O14B—C21B—C22B	161 (3)
C2—C1—C15—O3	−77.5 (4)		

### Hydrogen-bond geometry (Å, º)

**Table:**

*D*—H···*A*	*D*—H	H···*A*	*D*···*A*	*D*—H···*A*
O3—H3*A*···O5	0.82	2.11	2.821 (4)	145
C1—H1*A*···O15*B*	0.97	2.25	2.78 (2)	113
C3—H3*B*···O11	0.98	2.47	3.330 (6)	147
C4—H4*A*···O15*B*	0.98	2.54	3.12 (2)	118
C16—H16*A*···O3	0.96	2.62	3.222 (6)	121
C16—H16*C*···O15*A*	0.96	2.39	2.946 (7)	116
C37—H37*B*···O9^i^	0.96	2.39	3.239 (8)	147
C37—H37*C*···O15*B*^ii^	0.96	2.19	2.92 (2)	132

Symmetry codes: (i) *x*+1/2, −*y*+3/2, −*z*+1; (ii) *x*+1, *y*, *z*.
